# Urolithin A as a Potential Drug for the Treatment of Spinal Cord Injuries: A Mechanistic Study Using Network Pharmacology Approaches

**DOI:** 10.1155/2022/9090113

**Published:** 2022-04-22

**Authors:** Chao Mao, HaoPeng Luan, ShuTao Gao, WeiBin Sheng

**Affiliations:** Department of Spine Surgery, The First Affiliated Hospital of Xinjiang Medical University, Urumqi, Xinjiang 830054, China

## Abstract

**Objective:**

This research was focused to examine the potential targets, action network, and mechanism of urolithin A (UA) in spinal cord injury (SCI) management exploiting the network pharmacology (NP).

**Methods:**

We used the SwissTargetPrediction, PharmMapper, and TargetNet databases to obtain UA action targets. We searched the OMIM, GeneCards, CTD, and DrugBank databases to screen selected target genes for SCI treatment. The intersection of target genes between the UA and SCI databases was obtained by constructing Venn diagrams, which led to the identification of common druggable targets for the disease. The relationship network of the targets was built with Cytoscape 3.7.2, and the protein interaction network was analyzed with the STRING platform. The protein-protein interaction (PPI) network can be built on the STRING database. Gene Ontology (GO) function and KEGG pathway analyses of target intersections were completed with the DAVID 6.8 database. We constructed preliminary network targets for actions underlying UA-SCI interactions. Using the AutoDock software, we examined the molecular docking interactions between UA and its target proteins and further verified the mechanism of the action of UA.

**Results:**

We obtained 318 UA drug targets and 1492 SCI disease targets. We identified a total of 118 common UA-SCI targets. Based on the PPI analysis, we identified MAPK1, SRC, AKT1, HRAS, MAPK8, HSP90AA1, MAPK14, JAK2, ESR1, and NF-*κ*B1 as possible therapeutic targets. Enrichment analysis revealed that the PI3K-AKT, VEGF, and TNF signaling pathways could be critical for the NP analysis. Molecular docking indicated that UA had a strong affinity for docked proteins (binding energy range: −6.3 to −9.3 kcal mol^−1^).

**Conclusions:**

We employed an NP approach to validate and predict the underlying mechanisms associated with UA therapy for SCI. An additional purpose of this study was to provide a theoretical basis for further experimental studies on UA's potential in SCI treatment.

## 1. Introduction

Spinal cord injury (SCI) causes the most severe spine-related complications and can often result in functional limb impairment below the level of the injured segment [1]. SCI not only causes lifelong disabilities but also places a heavy socioeconomic burden on the patient's family [2]. The age-standardized incidence rate of SCI is 13 per 100,000 worldwide [3]. Notably, 80% of the patients diagnosed with SCI are young males (mean age: 29 years) [4]. Current SCI research is mainly focused on revealing the underlying mechanisms and development of drug interventions. Although there are many studies on SCI, finding the right and effective treatment is still challenging [5]. Therefore, exploring both effective therapeutics and molecular mechanisms of the disease are urgently warranted to aid treat SCI.

The blood-spinal cord barrier (BSCB) is functionally similar to the blood-brain barrier (BBB) that separates the spinal cord from plasma and significantly interferes with the entry of therapeutic drugs into the spinal cord [6]. Importantly, drugs that are capable of crossing the BSCB are critical for effective SCI treatment [7]. Urolithin A (UA), a natural compound commonly found in certain types of nuts and fruits, is also produced in the body by gut microorganisms utilizing the dietary ellagic acid (EA) [8]. In prior studies on Alzheimer's disease (AD), UA was shown to possess the capacity to pass through the BBB, and strong neuroprotective functions, as well [9]. Recent studies have further confirmed the neuroprotective efficacy of EA in both *in vitro* and *in vivo* disease models. With its potent antioxidant and anti-inflammatory effects, EA can counteract diverse types of neurotoxic and neurodegenerative conditions [10]. However, to date, there are no studies to show the mechanism of action of UA in protecting nerves from the SCI.

Network pharmacology (NP) exploits network topology mapping and bioinformatics to analyze the complex relationship between drugs, drug targets, affected signaling pathways, and diseases. In this context, the NP approach can improve the efficiency of target-oriented drug findings, thus significantly reducing the therapeutic development costs as well as providing new ideas for studying the drug-disease interaction mechanisms in clinical research [11]. Here, we used NP and molecular docking approaches to predict UA's potential targets and its mechanism of action in the SCI model and provide a reference for future investigations.

## 2. Methods

### 2.1. Screening of Related Targets

UA is a chemically simple compound. The SwissTargetPrediction database (https://www.swisstargetprediction.ch/) was applied to query UA's targets and names of relevant target genes. Then, the PharmMapper database (https://www.lilab-ecust.cn/pharmmapper/submitfile.html) was applied to restrict the species to humans, as well as the TargetNet database (https://targetnet.scbdd.com/) to identify potential target genes. Furthermore, we employed the UniProt database (https://www.uniprot.org/uploadlists/) to query genes corresponding to potential target proteins in screening for the active ingredient. Our results were derived from targeting UA through Excel searching and sorting. We used the search term “Spinal cord injury” in databases such as OMIM (https://www.omim.org/), GeneCard (https://www.genecards.org/), CTD (https://ctdbase.org/), and DrugBank (https://www.DrugBank.ca).

### 2.2. Obtaining the Anti-SCI Targets of UA

After merging and compiling the database search results, duplicate targets were removed. Intersections of the predicted target for the active component of UA and the retrieved results of SCI-related target genes were determined, and common targets were screened out as probable action targets of UA in SCI. We applied the Venny 2.1 (https://bioinfogp.cnb.csic.es/tools/venny/index.html) tool to map SCI-related targets and active ingredient targets in the form of Venn diagrams. Next, shared targets between the UA and SCI were noted as the potential target set for UA in SCI treatment.

### 2.3. Building a PPI Network

PPI networks are constructed from protein targets. that interact among themselves to execute certain biological processes such as cell signaling, regulation of gene expressions, metabolic pathways like energy production, and regulation of cell cycle checkpoints [12]. The STRING database v.11 (https://string-db.org/) was utilized to identify potential therapeutic targets of UA related to SCI pathology within the PPI network, setting the species type to “*Homo sapiens*” and a high confidence score (0.9) [13]. Following this, the Cytoscape (version 3.7.2) software (https://cytoscape.org) was used to visually output the PPI network.

### 2.4. KEGG and GO Analyses for Potential Therapeutic Genes for UA in the SCI

The DAVID 6.8, an open-source database (https://david.ncifcrf.gov/), was utilized for statistical analysis and graphical displays. This database has also been exploited for the development of online packages/tools such as Bioinformatics (https://www.bioinformatics.com.cn/) and OmicShare (https://www.omicshare.com/tools/) for mapping of GO-annotated and KEGG pathway enriched factors. We used these online platforms (adjusted *P* value cutoff of 0.05) to examine the biological processes (BP), molecular functions (MF), cellular components (CC), and signaling pathways linked to UA in SCI treatment.

### 2.5. Docking between UA and Targets

After determining UA-related targets and active compounds, we next screened for active compounds that could modulate the functions of those top 10 hub genes. A Sankey diagram (https://sankeymatic.com/) was plotted to visualize the “hubs in UA-bioactive compounds-top 10 hub genes.” Selected critical bioactive chemical compounds designated for low molecular weight ligands, and their respective great receptor proteins were subjected to the molecular docking assay. The original document of latent bioactive compounds related to UA may be publicly accessed through the PubChem compounds database (https://pubchem.ncbi.nlm.nih.gov/) and that of the top 10 hub genes (PDB format) through the RCSB Protein Data Bank (https://www.rcsb.org/). After performing the initial analysis with the AutoDock tool [14], the existing data format was converted into the PDBQT format using the PyMOL software (https://pymol.org/2/) for the docking assay [15]. As described elsewhere, a smaller molecular docking score of each interaction reflected a higher binding affinity of the ligand with the respective receptor protein(s) [15].

## 3. Results

### 3.1. UA- and SCI-Related Target Acquisition Results

The SwissTargetPrediction, PharmMapper, and TargetNet databases were used to determine UA-associated action targets. Totally, 314 UA-linked action targets were identified and retrieved from these databases in the end. All together 1492 targets for SCI were retrieved in OMIM, GeneCards, CTD, and DrugBank databases.

### 3.2. Determination of Effective UA Therapeutics for SCI

**Figure 1:**
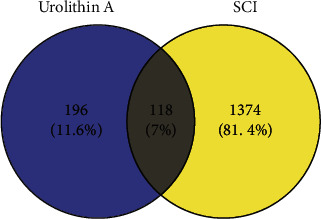
The Venn diagram illustrating the intersect of UA- and SCI-related targets.

The overlapping UA- and SCI-related targets were analyzed by constructing a Venn diagram to find promising therapeutic targets for UA in SCI treatment, which resulted in a total of 118 potential targets (Figure 1).

### 3.3. Building the PPI Protein Interaction Network

**Figure 2:**
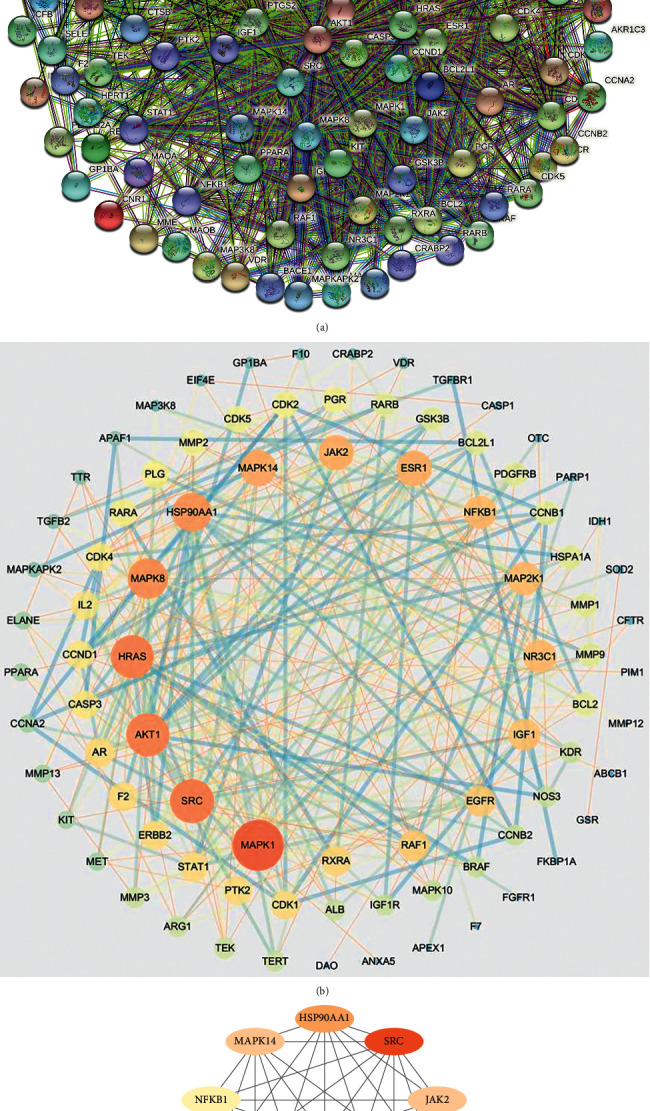
The PPI network identifying the top 10 hub genes for UA against SCI. (a) STRING-based PPI analysis construction. (b) Cytoscape-based PPI analysis construction (intensity of the node color is proportional to the amount of linked proteins). (c) Determination of the top-ranked hub genes for UA against SCI using the degree algorithm.

A PPI network within the STRING database was constructed by entering previously identified 118 potential targets for UA in relation to SCI pathology, and setting the species to “*Homo sapiens*” (Figure 2(a)), which incorporated 118 nodes and 341 edges. Then, the Cytoscape software was used to retrieve the names of those hub genes on the PPI network by constructing a “UA-active compounds-target genes-SCI” network (Figure 2(b)), where interconnecting lines between any two nodes predicted the presence of internode relationships. The greater the number of nodes was, the more the relationships were predicted.  Figure 2(c) describes the basic information on the top 10 hub genes (*MAPK1*, *SRC*, *AKT1*, *HRAS*, *MAPK8*, *HSP90AA1*, *MAPK14*, *JAK2*, *ESR1*, and *NF-κB1*) for UA in SCI treatment.

### 3.4. KEGG and GO Pathway Enrichment Analyses

**Figure 3:**
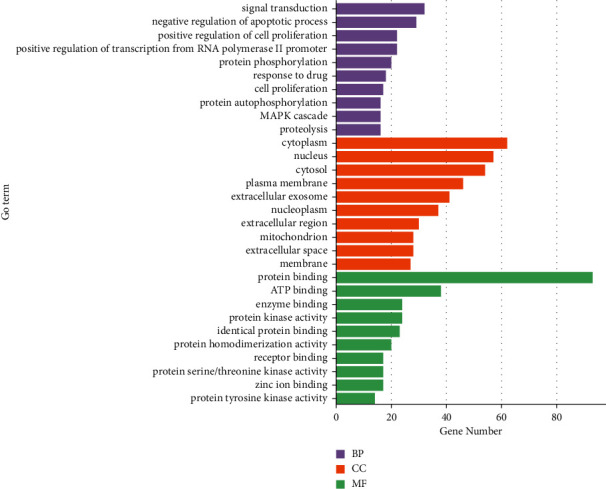
The bar chart of top 20 GO (BP, CC, and MF) enriched items.

**Figure 4:**
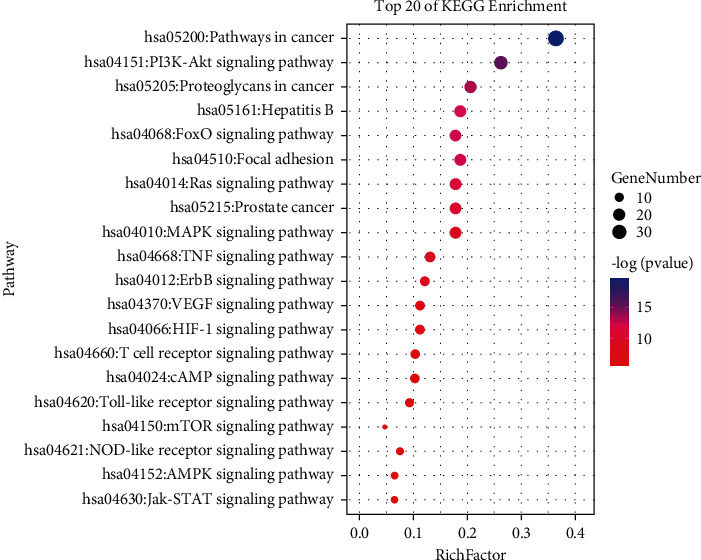
The analysis of the KEGG pathway of the UA-SCI common targets. The top 20 related pathways were visualized.

The ClusterProfiler was used to explore the GO database for the functional enrichment of pathways, such as biological process (BP), cellular components (CC), and molecular function (MF), which showed the enrichment of 123 GO terms associated with targets of UA against SCI. We then screened the top 20 GO terms (*P* < 0.05) (Figure 3). Further using the ClusterProfiler platform, we also performed a KEGG enrichment analysis to visualize the relationship of the UA target with cellular signaling pathways (cutoff *P*=0.05). A total of 148 pathways were found to be enriched in this analysis. The top 20 pathways thus identified, including the P13K-AKT, MAPK, TNF, VEGF, and HIF-1 signaling pathways, suggest that the pathways targeted by UA may modulate inflammatory responses and inhibit cellular apoptosis in SCI patients (Figure 4)

### 3.5. Molecular Docking Analysis

**Figure 5:**
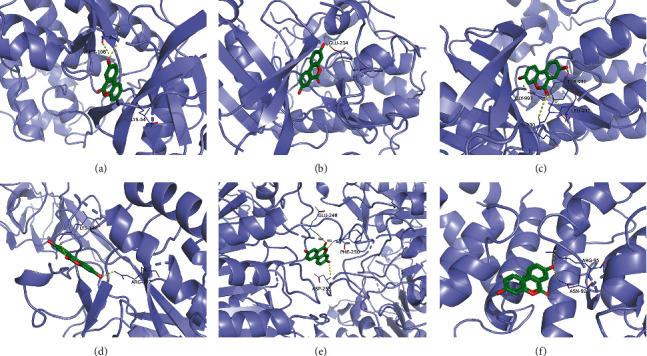
Docking of UA-related active compounds and prospective hub genes. (a) *MAPK1*, (b) *AKT1*, (c) *JAK2*, (d) *NF-κB1*, (e) *CASP3*, and (f) *BCL-2*.

Molecular docking analysis further confirmed the pathway prediction results revealing the existence of substantial interactions between the active ingredients of UA and their respective hub genes. Exploring the underlying mechanism of UA in SCI management might delineate the mechanistic regulation of targets such as MAPK1, AKT1, JAK2, NF-*κ*B1, CASP3, and BCL-2. Furthermore, this finding indicated the reliability of using the NP approach in parallel to the molecular docking for the target identification. The docking results showed that the minimum binding energy between UA and the ligand-binding sites in the receptor proteins MAPK1, AKT1, JAK2, NF-*κ*B1, CASP3, and BCL-2 was less than −5.0 kcal mol^−1^ (Figure 5). Moreover, molecular docking exhibited that UA had a strong affinity towards docked proteins (with binding energy ranging from −6.3 to −9.3 kcal mol^−1^), suggesting a tight binding interaction of UA with the target proteins.

## 4. Discussion

SCI is characterized by an abnormal change in the structure and function of the spinal cord and is directly or indirectly caused by a variety of pathogenic factors. This often leads to impairment or loss of sensory, motor, and neurological functions of the limbs below the injured segment [16]. SCI is classified as the primary and secondary injuries based on the mechanism of injury. A primary injury is characterized by the squeezing, traction, tearing, and shearing of the spinal cord tissue and associated blood vessels at the time of injury, leading to rapid cell death [17]. Secondary injury refers to a series of pathological reactions that occur after the injury, including neuronal death, axonal growth inhibition, ischemia and hypoxia, spinal cord tissue edema, and inflammatory reactions, resulting in severe nerve tissue damage [18, 19]. Thus, research on neuroprotective drugs that could potentially reduce or prevent further nerve injury is important for the restoration of nerve function following the SCI. Presently, the specific molecular mechanism underlying UA's effects on the SCI is not clear. Using NP methods, this study demonstrated the promising roles of UA for the treatment of SCI.

Using the STRING interaction network database, we constructed a target interaction network representing UA effects on the therapy of SCI. All together, 118 core targets were screened and selected. The NP analysis and molecular docking results predicted several key target genes, including *MAPK1*, *SPC*, *AKT1*, *HRAS*, *MAPK8*, and *NF-κB1* for UA in the SCI treatment. These six target genes may critically modulate the UA-based treatment of SCI. Previous studies have shown that traumatic SCI leads to the activation of NF-*κ*B and MAPK pathways [20]. Thus, blocking these signaling pathways could be promising in treating SCI. Liu et al. [21] and Xiao et al. [22] found that targeted suppression of NF-*κ*B and MAPK signaling was neuroprotection against the SCI. Abdelazeem et al. [23] reported that UA could suppress both NF-*κ*B as well as MAPK signaling pathways in bone marrow stromal cells by inhibiting the generation of reactive oxygen species. Others have also shown that UA could inhibit the NF-*κ*B nuclear translocation by blocking IKB*α* phosphorylation, thereby modulating the activity of NF-*κ*B under the diseased condition [24]. The MAPK pathway activation is important for the regulation of cell proliferation, differentiation, and apoptosis [25]. UA plays a neuroprotective role via modulating the p38 MAPK pathway. Kim et al. [26] has demonstrated that UA protects against apoptosis and inhibits the p38 MAPK pathway-associated inflammation.

AKT was shown to have a central role in the key target network for UA in the treatment of SCI. AKT is a serine-threonine kinase related to protein kinase C (PKC) [27], which is a direct downstream target of PI3K. Studies have shown that the abnormal PI3K pathway activation is detrimental to the pathogenesis of SCI. The PI3K protein family includes a series of intracellular enzymes and, when phosphorylated, activates AKT, which resides on the cellular membrane. AKT is released from the cell membrane into the cytoplasm or nucleus, depending on the signal transmission pattern [28]. Therefore, AKT phosphorylation can be used as an indicator to determine the PI3K activity. In recent years, the action of a PI3K-AKT pathway in SCI has attracted significant attention. The PI3K-AKT signaling mechanism regulates cytokine activation and has a critical role in the mechanisms of SCI [29]. Xu et al. [30] and Li et al. [28] have found that, in the course of SCI pathology, activation of the PI3K-AKT signaling could prevent neuronal apoptosis, regulate neuronal differentiation and cell migration; significantly promote the excretion of neurotrophin and intercellular adhesion molecules by astrocytes; reduce the expressions of chondroitin sulfate proteoglycan and glial fibrillary acidic protein; inhibit the formation of the glial scarring; and decrease the multiplication of astrocyte cells. Fu et al. [31] have found that UA can suppress PI3K phosphorylation and downregulate AKT expression in a dose-dependent effect, thereby inhibiting the secretion of TNF-*α*, IL-6, and similar cytokines. Once the central nervous system (CNS) is injured, the JAK/STAT3 pathway participates in gliosis and scar formation by regulating the differentiation of neural progenitor cells (NPCs) and activation of astrocytes in SCI tissues [32, 33]. Zheng et al. [34] have shown that the application of JAK2 inhibitors and regulation of inflammatory factors expression at an early phase of SCI can substantially reduce neuronal apoptosis and promote functional recovery. Liu et al. [35] has further reported that JAK inhibitors could block the JAK/STAT3 pathway in astrocytes after SCI, thereby inhibiting the formation of glial scars. During apoptosis, CASP3 is the most important terminal cleaving enzyme [36]. Endoplasmic reticulum (ER) stress following SCI can cause locomotor dysfunction, neuronal cell apoptosis, and suppression of the PI3K-AKT pathway [37]. Li et al. [37] reported that icariin could significantly inhibit the expression of apoptotic proteins induced by ER stress, such as CASP3, and exert protective effects on functional recovery and neuronal cell survival post-SCI. Apoptosis is a process of programmed cell death regulated by an intracellular signal transduction pathway [38]. SCI is often accompanied by apoptosis. BCL-2 can play an anti-apoptotic role, while Bax and CASP3 are directly involved in the induction and mediation of apoptosis. The Bax protein not only antagonizes the anti-apoptotic effects of BCL-2 but also promotes apoptosis by activating the subsequent enzymatic cascades involving the Caspase protease family proteins. Ye et al. [39] have found that UA increases the expression of anti-apoptotic BCL-2 proteins and decreases neuronal apoptosis by downregulating the miR-34 expression.

The KEGG pathway analysis explored that the major pathways to participate in SCI pathology contain the PI3K-AKT, VEGF, and TNF signaling pathways. PI3K-AKT is critical in neuronal regeneration and inhibition of neuronal apoptosis. This pathway also regulates a variety of cellular responses, such as cell cycle, stress, and apoptosis [37]. Two key apoptotic downstream proteins in the PI3K-AKT pathway are Bax and BCL-2. The expression of BCL-2 in the cell cycle-associated mitochondrial apoptosis is regulated by the PI3K-AKT signaling pathway [40]. Cheng et al. [41] have shown that promoting phospho-AKT could heighten the BCL-2 expression while reducing Bax and cleaved-CASP3 levels in SCI. Liu et al. [42] have shown that VEGF can increase the post-transplant survival rate of the bone marrow mesenchymal stem cells (BMSCs) in SCI patients. BMSCs may participate in the pathological reaction of blood vessels and nerve cells following the SCI by autocrine and/or paracrine VEGF signaling pathway promoting the migration of endothelial cells to form new microvasculature, which in turn improves the local microcirculation in SCI tissue. This mechanism can indirectly protect nerve cells from ischemia and hypoxic injury, as well. Additionally, the transfer of VEGF to SCI sites can promote the regeneration of axons after injury [43]. TNF-*α* increases immediately in response to CNS damage (including SCI), skin, muscle, and bone injuries [44]. Peng et al. [45] have found that TNF-*α* inhibits the differentiation of BMSCs into NPCs and significantly restored neurological function in SCI rats. Han et al. [46] reported that BMSC transplantation could markedly decrease expressions of TNF-*α*, IL-1*β*, and IL-6, promote axonal regeneration, and help in locomotor function improvement. Furthermore, glial scar formation and cavities were reduced in the transplanted rats.

## 5. Conclusions

In summary, this study utilized the NP methodology to explore the role and mechanism of UA for treating SCI. The results of this study suggest that UA may regulate inflammation and inhibit apoptosis through multiple pathways, contributing to improved functioning of damaged nerves in SCI. There were 118 therapeutic targets in UA-SCI target screening, among which *MAPK1*, *AKT1*, *MAPK8*, *MAPK14*, *NF-κB1*, and *CASP3,* were the hub genes. We also identified critical UA pathways associated with SCI treatment, including the PI3K-AKT, VEGF, and TNF signaling pathways. Here, we employed the NP approach to study the mechanism of UA for treating SCI and to provide a basis for future studies. Our predicted disease targets for UA components closely matched with those in the existing literature, indicating that the use of a combination of NP and molecular docking to study the treatment mechanism could be useful and accurate. However, this study was mainly grounded in bioinformatics, and further studies with animal models and/or cell experiments are needed to verify the main regulatory targets of UA in SCI therapy.

## Data Availability

The data sets generated and analyzed during the current study are not publicly available due to restrictions on ethical approvals involving patient data and anonymity but can be obtained from the appropriate authors as reasonably required.

## Supplementary Materials

Supplementary Materials Raw data: details of the genes.
