# Supplementary material for: Urolithin A as a Potential Drug for the Treatment of Spinal Cord Injuries: A Mechanistic Study Using Network Pharmacology Approaches
Source: Evid Based Complement Alternat Med. 2022 Apr 22;2022:9090113. doi: 10.1155/2022/9090113 (PMC9054438; doi:10.1155/2022/9090113)
Supplement: Supplementary Materials — Raw data: details of the genes. [file 9090113.f1.pdf]

UA-related targets	swiss	XDH	SCI-related targets	genecard	GFAP	≥12
		ESR1			IL6	
		ESR2			TNF	
		CA12			SOD1	
		CA9			SMN1	
		CA13			TP53	
		MAOA			IL10	
		CA6			IGHMBP2	
		CA14			SMN2	
		CA5A			DARS2	
		GPR35			NF1	
		CDK4			CCL2	
		CCND1			NGF	
		PDGFRB			F2	
		FLT4			IFNG	
		INSR			TARDBP	
		CCNA1			TLR4	
		CCNA2			CD40LG	
		CDK2			SETX	
		SRC			C9orf72	
		PTK2			VAPB	
		PLK4			TRPV4	
		TEK			CTNNB1	
		AKT1			DCTN1	
		AURKA			BDNF	
		MAP3K8			AKT1	
		EPHB4			GARS1	
		HSPA1A			MTHFR	
		NUAK1			NOS2	
		SQLE			FUS	
		FGR			MAPT	
		LYN			AR	
		DAO			COMT	
		CA5B			AQP4	
		GSR			MNX1	
		CA7			BICD2	
		AURKB			MBP	
		AKR1B1			VANGL1	
		CA1			GDAP1	
		CA4			NTRK1	
		EGFR			CHAT	
		CA2			COL2A1	
		IGF1R			GDNF	
		KDR			COL1A1	
		PLK1			ALS2	
		MET			CACNA1A	
		CSNK2A1			ICAM1	
		GSK3B			SHH	
		BRAF			DYNC1H1	

ERBB2	ATXN2
BACE1	TLR2
CA3	PTEN
SRD5A1	NF2
HSD17B3	CCND1
AKR1C3	S100B
AKR1C1	IL1B
KCNA3	CASP3
HSP90AA1	NEFL
NFKB1	GRIN1
IDH1	BSCL2
STS	TTR
MCL1	TGFB1
BCL2	HLA-DRB1
PLG	CXCL8
PARP1	ATXN3
TNKS2	ATP7A
TNKS	APP
ERN1	SPG7
PTGS2	PLEKHG5
CDK5	MTOR
CDK5R1	HSPB8
CES2	MMP9
KCNA5	EP300
ABCB1	MTHFD1
APEX1	ATXN1
PTPN1	MPO
PON1	MPZ
DYRK1A	POLG
GSTA1	PLP1
KCNMA1	EGFR
ALDH2	ENG
FNTA FNTB	SH3TC2
CDK1	DARS1
CCNB1	SPG11
CCNB2	RET
CCNB3	PTCH1
CDK6	ERCC6
NQO1	BRAF
TNNT2	MYC
TNNI3	FGFR3
TNNC1	TH
CBR1	PMP22
BCL2L1	SLC5A7
NAT1	DKK1
MIF	NEFH
CNR1	SQSTM1
CNR2	INS
PTP4A3	LMNA
TERT	PRX

	NOX4	SMARCB1
	FLT3	FGFR1
	SYK	VCP
	HSD17B2	PIK3CA
	ABCC1	SPP1
	CFTR	SOX9
	DHODH	APOE
	PIM1	ATXN7
	RAF1	ALB
	F2	SMARCA4
pharmmap	ABL1	CHCHD10
	ABO	LMNB1
	ADAM17	ENO2
	ADAM33	THBD
	ADH5	NRAS
	ADK	RYR1
	AGXT	FGFR2
	AK1	REEP1
	AKR1C2	SCN8A
	ALB	IGF1
	AMD1	PRPH
	AMY1A	SPTAN1
	AMY1B	SMAD3
	AMY1C	ARSA
	AMY2A	ASAH1
	ANXA5	MTR
	APAF1	HSPG2
	AR	TSC2
	ARG1	FAS
	ARHGAP1	SCN9A
	ARL5A	VANGL2
	Arl5b	ZIC1
	ATIC	EPRS1
	BCAT2	DRD2
	BCHE	ELN
	BHMT	ADA
	BIRC7	CRP
	BMP2	MFN2
	BST1	GALNS
	BTK	MAG
	CASP1	KIF1B
	CASP3	BRCA1
	CBS	MORC2
	CCL5	TRIP4
	CDK2	VEGFA
	CDK5R1	STAT3
	CES1	DNAJB2
	CFB	PPARG
	CFD	GJB1
	CHEK1	TK2

CHIT1
CLK1
CRABP2
CRYZ
CTNNA1
CTSB
CTSF
CTSK
CTSS
CYP19A1
CYP2C9
DCK
DCXR
DHFR
DPEP1
DPP4
DTYMK
DUSP6
DUT
EEA1
EIF4E
ELANE
ERI1
ESRRG
F10
F11
F7
FABP3
FABP7
FECH
FGFR1
FGG
FKBP1A
FKBP1B
FKBP3
FNTA
GART
GCK
GMPR
GP1BA
GPI
GSTA3
GSTM1
GSTM2
GSTP1
GSTT2B
HADH
HCK
HDAC8
HEXB
FIG4
PDYN
VWF
KIF1A
CREBBP
HNRNPA1
GJA1
MTRR
FOS
SYP
TNFRSF1A
SMAD4
FMR1
LEP
FXN
CAPN3
C3
VRK1
NAGLU
KRAS
OPTN
SLC2A1
MME
LITAF
IDH1
SDHB
SERPINE1
PSEN1
HRAS
NAIP
PTPN11
NOS3
GLE1
NLRP3
SNCA
ATL1
SDHD
TSC1
IL4
HLA-B
NOG
NTRK2
IL2
NGFR
GNAS
TGFBR2
MECP2
CDH1
ARSB
CAT

HINT1
HMGCR
HNF4G
HNMT
HPN
HPRT1
HRAS
HSD11B1
HSD17B1
HSPA8
IGF1
IL2
IMPDH2
ISG20
JAK2
JAK3
KAT2B
KIF11
KIT
LCK
LTA4H
LYZ
MAN1B1
MAOB
MAP2K1
MAPK1
MAPK10
MAPK14
MAPK8
MAPKAPK2
MME
MMP1
MMP12
MMP13
MMP16
MMP2
MMP3
MMP8
MMP9
MTAP
MTHFD1
NMNAT1
NOS3
NR1H2
NR1H3
NR1H4
NR1I2
NR3C1
NR3C2
NT5M
TBK1
AARS1
TGFB2
HSPB1
FLVCR1
SPAST
GJB2
SDHC
CD46
MATR3
MT-ATP6
CNTNAP2
STAT1
UBQLN2
GALC
MIR21
ERBB2
HLA-DQB1
VIM
CHMP2B
IL17A
CTLA4
NTF3
SLC1A2
FA2H
PRNP
HLA-DQA1
ITGB2
ASCC1
FOXP3
SIGMAR1
TWNK
TBP
SCN11A
COL9A3
CDK4
COMP
STXBP1
FGF2
SPTLC1
PNPLA6
CELSR1
FLT1
LZTR1
PHGDH
SCRIB
PAX6
GLB1
TTN
PNKP

OAT	TREM2
OTC	IL1A
PAK6	PON1
PCK1	CALCA
PDE3B	OLIG2
PDE4B	HTR2A
PDE4D	ERBB4
PDE5A	HLA-A
PDPK1	F8
Pfkfb1	SYNE1
PGF	KIF5A
PGR	WASHC5
PKLR	COL9A2
PLA2G2A	RELA
PNMT	CSF3
PPARA	CNTF
PPARD	CSPG4
PPP1CC	PLA2G6
PPP5C	FGF8
PRKACA	GJC2
PYGL	PDCD10
RAB5A	DES
RAB9A	F7
RAP2A	GRM1
RARA	DNMT1
RARB	ITGAM
RARG	U2AF1
RBP4	CXCL10
REN	NOS1
RFK	MMP2
RNASE3	MIR146A
RORA	KCNA1
RXRA	NOTCH3
RXRB	UCHL1
S100A9	CFI
SDS	PDE4D
SELE	RTN4R
SETD7	PLG
SHBG	CDKL5
SOD2	ANG
SORD	ATRX
SPR	RTN4
ST14	ZFYVE26
STAT1	MEN1
SULT1A1	TGFBR1
SULT1E1	ISL1
SULT2A1	PPARGC1A
SULT2B1	SPART
TAP1	CCR5
TGFB2	CDKN1B

TGFBR1
TGM3
THRA
THRB
TK1
TPH1
TPI1
TRDMT1
TTPA
TTR
TYMS
UAP1
UCK2
VDR
WARS1
XIAP
YARS1
HEXB
PRKACA

AQP1
ARG1
SOX10
MARS1
SCN1A
GM2A
CCR6
TCN2
MOG
FKRP
FGA
RAB7A
KRIT1
MN1
CD4
NOTCH2
GRIN2B
RETREG1
DICER1
HGF
PRKCG
PRF1
KCNQ2
PLEC
MAP2K1
IGF2
MUSK
C4A
KLK8
EPO
SLC25A22
AFP
WNK1
IL7R
IDUA
ACTA1
KCNJ10
BMP4
CACNA1G
TERT
MEFV
CCT5
NDUFS4
BMP2
SDHA
CAV1
PTGS2
COL1A2
NES
CD44

F9
SPTBN2
MAPK10
HPRT1
SLC6A4
MUC1
MIR126
PES1
SUFU
CD36
IL18
CFH
PRPS1
SACS
SLC12A6
FKTN
CDKN1A
RTN4IP1
CCM2
SELENON
COL11A1
FGD4
BRCA2
ITGB3
TGFB3
LRSAM1
MT-ND4
F5
PDGFB
PRICKLE2
AKT3
PROM1
SERPINH1
ACAN
ACVRL1
GRIA3
SMARCC2
TSEN54
IL1R1
INPP5E
FBXO38
FBN1
SLC18A3
MT-ND1
NKX2-1
MAPK1
RUNX2
ACHE
HFE
TBXT

FCGR2A
MYCN
APC
MIR125A
MT-ND6
NOD2
H2AC18
AFF2
ENPP1
VHL
EDN1
TIMP1
ACE
ACTB
EGF
GLS
MIR17
ALDH18A1
F3
TNFSF13B
DMD
ALDH5A1
GLI3
RAI1
CSF2
TNFRSF1B
SERPINA3
GUSB
TBCE
RELN
FHL1
SERPINC1
SURF1
SBF2
RPL34
GAMT
HARS1
SPTLC2
ARID1B
ACP5
COL4A1
TOR1A
ERBB3
NOTCH1
MPV17
ARX
IL5
PMS2
NPY
PTPN22

CCR1
GBA
EPHA4
VCAM1
IL1RN
GRHL3
PRKAR1A
CCL3
NCAM1
DHCR7
RASA1
GNAQ
EXOSC3
PIGQ
BCOR
EMD
IDS
H19
YARS1
BGLAP
IL13
WNT1
CNTNAP1
MAP2
HTT
THBS2
FASLG
NPC1
HSPB3
GAP43
ALMS1
MT-CYB
CXCR4
TUBB4A
ATP1A3
MIR34A
PDGFRA
PRL
CPT2
MMP1
KMT2D
AARS2
IFNB1
MIR144
POMC
B3GALT6
TUBB3
SCN10A
CST3
PLCB1

LIG4
CFTR
EGR2
LFNG
AGRN
HSP90AA1
SERPINA1
ITPR1
RARS2
IFIH1
KCNC3
KDM1A
CASP8
TAC1
HSPA4
TUBA4A
COX10
WWOX
SLC1A3
MMP3
F10
CSF1
SCN4A
ELP1
IARS2
DAO
GH1
MYD88
LRP2
ALK
MSX2
SLC52A3
CXCL1
CNP
PKD1
BCL2
NTN1
POMGNT1
OCLN
SYT1
JAG1
F13A1
SDHAF2
AFG3L2
VDR
SCN2A
CPLANE1
NFIA
IFNA1
FN1

CXCL12
AIF1
HEXA
B2M
FCGR3B
MIR9-1
CCL5
SMARCE1
C12orf65
BMP1
MVK
GNAO1
GPT
SST
TDP1
FGF14
IBA57
G6PD
GP1BA
IL2RA
CD34
SOST
DNM2
CYCS
TFG
DDR2
RIPPLY2
MSH2
ADA2
MMP12
SCN5A
CCDC88C
PRKN
NLRP1
SLC6A3
SOX3
H3-3A
BCL2L1
PON2
DYSF
GAPDH
LGALS3
TCF20
MIR221
EPAS1
CDH2
LAMA2
ADAR
SELP
KIF7

ITGB1
GSN
EXOSC8
FOLH1
MGMT
SLC25A46
HCRT
HMOX1
CACNA1S
DNM1
CASP9
SOD2
APOA1
FANCB
PRODH
CTDP1
MIR223
LRRK2
DRP2
APEX1
ADSL
DAG1
EPHB2
TLR3
TBX1
SELE
SIL1
APOH
HSPD1
GCH1
GLUL
MYH7
PLAT
LRP5
HIF1A
PNPO
SLC17A5
IL17RA
LHX3
KIT
NTF4
MESP2
MT-TK
CD8A
HBB
GLI2
PLD3
PECAM1
ARID1A
CLIP2

NKX2-8
IL17F
PDCD1
TSEN2
FLNA
PAX2
CCL4
CLEC7A
MT-ND3
KDR
SOX2
CNR1
MYOD1
GRN
REN
MAOA
GMPPB
TMEM231
CRYAA
TPI1
CDKN2A
CSPP1
CP
COL6A3
HNF1A
CREB1
JUN
NFE2L2
OMG
TREX1
NSD2
WFS1
MIR145
GEMIN4
HECTD4
NIPBL
MAPK8
ABCB1
ATXN10
CRLF1
B3GALNT2
ERCC4
PLAU
TUBB2B
CALB2
ERCC2
TRPV1
IL23R
HNF4A
GLRX5

PHOX2B
GABRG2
PARD3
CASP1
ESR1
MPDZ
NRG1
FBLN5
COL10A1
PLOD1
STAT4
TSEN34
ITGB4
MAP1B
POMT1
OPRM1
IDH2
LEPR
CD79A
PDGFRB
RHO
TUBB
CR1
CHRNG
MIF
PRDM10
SOX11
SCARB2
SCN1B
UNC119
SLC18A2
WNT5A
AP4M1
ATN1
GATA1
GSR
ASCL1
BAG3
MRAP
FGF23
SMAD1
PRICKLE1
KCNA2
CXCL13
CEP290
CNTN2
STUB1
TRIM2
MMP13
BMP7

DHFR
PHF6
RPS27A
LCN2
FLNB
SNAP25
MIRLET7D
TMEM67
PAX1
PVALB
PSTPIP1
MBL2
CRYAB
LTA
CDK1
SFTPC
OFD1
MIR210
ACTA2
BOLA3
WNT3A
TIMP2
PFN1
MYPN
DPF2
CBS
COL5A1
SLC4A1
RBP3
TFRC
KITLG
EXOSC9
PEX6
RARB
DNA2
NDUFAF3
HEPACAM
MIR10A
RBM8A
MAPK3
NFASC
CD40
SALL4
RAPSN
FLII
RRM2B
XDH
IL12A
PANK2
MB

CDC42
NEK1
CASK
SNX14
CDKN2B
SEMA3A
APAF1
RERE
KNG1
KIF21A
ALDH1A2
ICOSLG
GRIA2
COL7A1
HTR1A
GJB6
KCND3
CCR2
TLR9
GAD1
DOK7
SAMHD1
AIFM1
TACR1
IGFBP3
MAOB
FOLR1
LDLR
SMAD7
RB1
NPPB
LBR
NEFM
ELANE
PSAT1
EMX2
AP1S2
PINK1
GAN
RNASEH2C
PAX3
PARP1
KCNT1
NDRG1
NDUFS2
TNFAIP3
ADRB2
COL6A2
CYP7B1
TCOF1

NDUFAF2
IL3
EDNRB
LEMD3
C2CD3
CHD7
ELOVL4
LPAR1
FANCA
FZD3
PIK3C2A
SIRT1
BMP6
COX15
EPX
MT-ND2
HLA-DPB1
CYP1A2
ARHGEF10
RPGRIP1L
C19orf12
MTM1
CYP27A1
RAF1
DNMT3A
TF
PAPPA
GATA2
NRXN1
NDUFV1
BAZ2B
PARK7
GLA
RNASEH2A
RNASEH2B
SDHAF1
SLC6A5
MIR93
DPYSL5
UCP2
MMUT
WRN
NUBPL
NAT2
GRP
CD27
TCTN2
DPH1
RAD51
MYLK

MIR142
CHRNE
MGME1
BCS1L
SGCE
PTGS1
IRF5
NDUFS3
NKX2-2
SYNJ1
LMOD3
SMAD6
AMPH
CC2D2A
SOX5
HSPA5
TMEM216
GLRA1
ADIPOQ
VPS13A
FKBP1A
LIF
TEK
ADCY10
UBA5
MIR15B
TNFRSF11B
PHEX
ITGA4
PMM2
TYROBP
RICTOR
PSAP
COL11A2
NSD1
GNPTAB
FAT4
TSPO
MECOM
DLD
COQ2
RASA2
COL5A2
TKT
DLL1
PTH
IQSEC2
SNRPB
KIAA1109
EGR1

NPM1
PTCH2
TMEM43
DNM1L
PCNA
SUCLG1
IRAK4
SLIT2
RHOA
MT-CO3
SETBP1
DMPK
ALX4
SALL1
PLA2G2A
TLR1
DCTN2
LOX
TNFSF11
EHMT1
SCN3A
COX5A
PF4
HES7
ZIC3
KIDINS220
FOXG1
MALAT1
NPC2
SERPINI1
LIMK1
CXCR2
C4B
MET
VTN
GBE1
NPPA
SRC
GLDC
L1CAM
PTPRC
RMRP
KMT2A
WT1
DVL1
RTN2
CDK2
DDC
HOXA1
VIP

NALCN
PMPCA
PGR
BECN1
CBSL
PPARA
ARSH
SUMF1
ATP2B3
PPP2R2B
HDAC6
CDK5
OLIG1
ERAP1
GRID2
SLC12A5
FOXRED1
NDUFAF5
ZIC2
HNF1B
PIK3CG
DDX41
PMP2
MTFMT
ACVR1
TLR5
ACY1
ADGRG6
NDE1
NDUFA1
GRIA1
POMGNT2
MKS1
COLQ
NBN
ADORA2A
CTBP1
IL15
AUTS2
HTRA1
DCAF8
ANGPT1
STAR
HP
ITGAL
ITGA2B
ATM
FOXO3
GLI1
TNFRSF11A

	JAK2
	PTGDS
	TGM6
	ALX3
	MIR15A
	TYR
	KLK6
	NDUFS8
	CRABP2
	CD28
	ERCC1
	NDUFS1
	CLDN11
	IL7
	SMC1A
	MDM2
	COASY
	AIMP2
	DYM
	ABCA1
	SMAD2
	ABCD1
	ANXA5
	CX3CR1
omim	NINJ2
	SCA18
	SMAR
	UBA1
	AC
	ASC1
	ASPRS
	CMT4A
	DARS
	DHTR
	DNCL
	FTDALS2
	GARS
	HRG4
	HSJ1
	KIAA0699
	KIAA0720
	MNK
	p50
	SMA1
	SMNA
	SPDA1
	SRBP
	UBE1
	VAPC
	VRNF

VROAC
ALS16
ALS8
CMT2K
DNECL
DSMA4
GXP1
HBSL
HMSN2C
HSPF3
IMD13
MK
SMA2
SMABF1
SMABF2
SMAD1
SMAJ
SMALED2A
SMAPME
TFM
WSS
A1ST
CMT20
CMT2C
CMT2D
CMTRIA
CMTRIC
DSMA2
DSMA5
IMMD
MDCDC
NFNS
OHS
SBMA
SMA3
SMALED2B
HMN5A
KD
MRD13
SMA4
SMAX2
SMAX3
SPSMA
AMCX1
SMAJI
SMALED1
SMAX1
SSQTL1
SMAL
VEXAS

		HYSP1	
		BCYM3	
		LBSL	
		XBP1	
		DDIT3	
		PPP1R15A	
		MAP1LC3B	
		ATF4	
		ATG5	
		AMBRA1	
		P2RX7	
		AKT1S1	
		GSK3B	
		AGT	
		BAX	
		NFKBIA	
		SREBF1	
		GPX1	
		ID1	
		INS1	
		LPL	
		CCNA2	
		MYBL2	
		GFAP	
		CYP3A4	
	CTS	GADD45A	≥20
		CYBA	
		NFKB1	
		BAD	
		PRKCB	
		FOXO1	
		TJP1	
		MMP10	
		NCF1	
		AGER	
		ITGA5	
		CYBB	
		ABCC2	
		RIPK3	
		JAK1	
		ALPL	
		HMGB1	
		PLK2	
		CDKN1C	
		TNFAIP2	
		LTB	
		CDH5	
		RPS6KB1	
		SELL	
		DIO2	

STAT6
BIRC5
ISG20
THBS1
DHRS3
IL12B
BNIP3
CCN2
IER3
TNFSF10
AKAP12
CCNB1
EIF2S1
SP1
IL1R2
LIPG
SOCS3
EIF4E
MARCKS
TXNIP
FBXO32
HSPA2
MARCKSL1
CD83
ABCG2
SLC2A4
FOSL2
MT1
ALOX5
TMPRSS2
FOSL1
IRF7
MAPK9
IGF1R
ATF3
TFAM
SOD3
JUNB
CD86
GSTP1
GNB1
ANXA3
SMPD1
LAMC1
LBH
CD68
NR3C1
CASP7
ATP2A2
FASN

TOP2A
MGST1
PSMB9
CEBPD
PTK2
ADGRE1
SDF2L1
FKBP5
LTF
MAPK14
LBP
HSP90B1
E2F1
CFLAR
CEBPB
INSIG2
PKM
IL17RD
ETS2
PLA2G7
BID
TRIM63
IFIT3
EIF2AK3
ENO1
ABCC1
CDK9
CCN1
CAPN2
ID2
EGLN3
NRF1
SGK1
MUC5AC
PLPP3
PRDX3
BAK1
IKBKB
TIMM17A
HD
EIF4EBP1
IFIT1
KLF7
PER1
CCND2
TRP53
COX1
IL1RL1
PRKAA1
CX3CL1

AVP
DGAT2
NUAK1
IL6ST
CXCL2
RXRA
PRRC2C
CDC25A
HMGCR
DDIT4
CTSD
ILK
SFTPB
PRKCA
FBXO15
TIPARP
MACF1
CITED2
LPIN1
IFI35
NRP1
NFE2L1
CXCL5
NR1D1
PGK1
PIM1
GSTM1
ADAMTS1
ENPP2
HDAC1
OAS1
IL4R
NFIL3
FST
NQO1
FMO1
IGFBP2
LGALS1
PLA1A
PTGES
G0S2
SPRY1
CENPU
EMC3
CDKN2D
SLC7A11
HNRNPAB
SLC16A1
NFKBIE
FSCN1

SERPINB5
ZFP36
GABARAPL1
ALAS1
GOT1
ACE2
EDNRA
GDF15
THRSP
OTC
PAK1
VEGFD
SZRD1
CGB3
PDGFA
CTSL
DUSP1
PLA2G4A
RARA
ANGPT2
LY6E
CYP1A1
CASP4
CYP2E1
HSPA1A
PTMA
RBL1
SLC7A5
RGS2
BHLHE40
RAB31
MSR1
ITGA3
MYH10
TNFAIP8
KLF6
ATF6
CHUK
ATP5A1
SMC4
POR
CCL20
CCNG1
ANXA2
ATP5F1B
MT2
IKBKG
NUDT6
S100A8
APOB

CRY1
PPARGC1B
CCNE1
DST
HLA-DRA
RBL2
UTP15
ABCB11
BCAR3
KCNMA1
MAPKAPK2
ALOX5AP
ABCC3
ADORA2B
GCLC
MKI67
CKM
CCNB2
HBEGF
PODXL
TNFRSF21
LCAT
TXNRD1
MYB
PLTP
DGAT1
GEM
SCARB1
SCD
VASP
PDK4
COX4I1
AREG
FEN1
PIK3R1
TRMT61A
ABHD11
FAM171B
MRPL17
TOP1
LIFR
MAP3K8
ACACB
PRKCZ
MRPL19
CCNF
SLC25A24
INHBB
THBS3
CTSB

ACTN1
IRS1
MRC1
RGS16
PRKCD
PPP3CA
CYP2B6
S1PR1
CXCL14
MAD2L1
RGS4
OCIAD2
GPAM
NCF2
RUNX1
SESN1
BACE1
KLF10
NOX1
PTBP2
AKR1C3
NEO1
ASNS
E2F8
LRP1
LAMC2
ADIPOR1
UQCRFS1
RAN
COL3A1
FOXM1
SKP2
BCL3
TRIB3
RFK
SRXN1
OAS3
NR4A1
GCLM
WSB2
CFB
KBTBD11
LHFPL6
SERPINB2
DYNLT3

UA∩SCI	ABCB1
	ABCC1
	AKR1C3
	AKT1
	ALB
	ANXA5
	APAF1
	APEX1
	AR
	ARG1
	BACE1
	BCL2
	BCL2L1
	BMP2
	BRAF
	CASP1
	CASP3
	CBS
	CCL5
	CCNA2
	CCNB1
	CCNB2
	CCND1
	CDK1
	CDK2
	CDK4
	CDK5
	CFB
	CFTR
	CNR1
	CRABP2
	CTSB
	DAO
	DHFR
	EGFR
	EIF4E
	ELANE
	ERBB2
	ESR1
	F10
	F2
	F7
	FGFR1
	FKBP1A
	GP1BA
	GSK3B
	GSR
	GSTM1
	GSTP1

HMGCR
HPRT1
HRAS
HSP90AA1
HSPA1A
IDH1
IGF1
IGF1R
IL2
ISG20
JAK2
KCNMA1
KDR
KIT
MAOA
MAOB
MAP2K1
MAP3K8
MAPK1
MAPK10
MAPK14
MAPK8
MAPKAPK2
MET
MIF
MME
MMP1
MMP12
MMP13
MMP2
MMP3
MMP9
MTHFD1
NFKB1
NOS3
NQO1
NR3C1
NUAK1
OTC
PARP1
PDE4D
PDGFRB
PGR
PIM1
PLA2G2A
PLG
PON1
PPARA
PTGS2
PTK2

RAF1
RARA
RARB
REN
RFK
RXRA
SELE
SOD2
SRC
STAT1
TEK
TERT
TGFB2
TGFBR1
TPI1
TTR
VDR
XDH
YARS1
